# Antimicrobial resistance patterns, virulence genes, and biofilm formation in enterococci strains collected from different sources

**DOI:** 10.1186/s12879-024-09117-2

**Published:** 2024-03-04

**Authors:** Maryam Ghazvinian, Saba Asgharzadeh Marghmalek, Mehrdad Gholami, Sanaz Amir Gholami, Elham Amiri, Hamid Reza Goli

**Affiliations:** 1 Molecular and Cell Biology Research Centre, Faculty of Medicine, Mazandaran University of Medical Sciences, Sari, Iran; 2 Department of Medical Microbiology and Virology, Faculty of Medicine, Mazandaran University of Medical Sciences, Farah Abad Blv, Khazar Square, Sari, Mazandaran Iran

**Keywords:** Enterococci, Antibiotic resistance, Biofilm formation, *Esp*, *Ace*, *efaA*

## Abstract

**Background:**

Currently, antibiotic-resistant strains of *Enterococcus* are considered to be one of the critical health challenges globally. This study aimed to investigate the antibiotic susceptibility pattern, biofilm formation capacity, and virulence genes of enterococci isolated from different sources.

**Methods:**

In this cross-sectional study, environmental and fecal samples were collected from the hospital environment, volunteers, and hospital staff from October 2018 to August 2019. The isolates were identified by morphological and biochemical tests (gram staining, catalase, bile resistance, esculin hydrolysis, carbohydrate fermentation, growth in 6.5% NaCl, Pyrrolidonyl arylamidase, arginine dehydrolase), and PCR for *ddl* gene. An antimicrobial susceptibility test was performed by the standard disk agar diffusion method according to the Clinical and Laboratory Standards Institute (CLSI) guidelines. Quantitative microplate assays were used to assess biofilm production. The bacterial DNAs were extracted by alkaline lysis method and polymerase chain reaction technique was used detect the e*sp*, *ace*, and *efaA* virulence genes.

**Results:**

Out of 145 isolates, 84 (57.9%) were identified as *E. faecalis* and 61 (42.1%) as *E. faecium*. Resistance to kanamycin and quinupristin-dalfopristin was 82.1% (69/84) and 85.7% (72/84), respectively, in *E. faecalis* isolates. Out of 61 *E. faecalis* isolates, 38 (62.4%) were resistant to kanamycin. Among the *E. faecalis* isolates, *esp* was the most dominant virulence gene (73.80%), followed by *efaA*, and *ace*, which were detected in 60.71%, and 30.95% isolates, respectively. In total, 68.27% of the strains were biofilm producers. Further, *esp* and *efaA* genes were more frequently found among *E. faecalis* strains with moderate and strong biofilm biomass.

**Conclusions:**

According to the findings of our study, enterococci strains isolated from different samples possess distinctive patterns of virulence genes. The *esp*, *ace*, and *efaA* genes were more prevalent among *E. faecalis* than *E. faecium*. Besides, the high level antibiotic resistance of normal flora and environmental enterococci strains is alarming the researchers.

**Supplementary Information:**

The online version contains supplementary material available at 10.1186/s12879-024-09117-2.

## Background

*Enterococci* are commensal organisms responsible for hospital-acquired infections in immunosuppressed patients [1]. Sources of infection are diverse, whereas these organisms may be transferred from environmental sources to animals and humans [2]. *Enterococcus faecalis* and *Enterococcus faecium* are predominant Gram-positive cocci in human clinical samples [1, 3]. Both organisms can be virulent to humans, but *E. faecalis* is more prevalent than *E. faecium* [4]*.* They are able to acquire new antibiotic resistance genes through a variety of mechanisms, which complicates the treatment of infections caused by these organisms [3]. However, *E. faecalis* and *E. faecium* are naturally resistant to clindamycin, trimethoprim-sulfamethoxazole, and gentamicin (low-level resistance) [5]. Furthermore, they can withstand all the antibiotics used to treat human infections [6]. However, a major concern is the emergence of vancomycin and teicoplanin-resistant organisms [6, 7]. Moreover, biofilm formation is recognized as a key factor in the development of enterococcal infections [8]. Biofilm can tolerate antimicrobial concentrations 100–1000 times greater than those needed to kill planktonic cells [9]. Biofilm-associated infections are difficult to treat because bacteria living in biofilms are resistant to antibiotics, environmental stress, and phagocytosis [10]. Microorganism adhesion to host cell surfaces is critical for the pathogenesis of infections and biofilm formation [8]. The most important virulence factors in *Enterococci* include the collagen-binding protein (*ace*), *E. faecalis* endocarditis specific antigen (*efaA*), and enterococcal surface protein (*esp*) [11, 12]. Ace, EfaA, and Esp are adhesion proteins that have an important role in adhesion to eukaryotic cells and surfaces along with the colonization of host tissues [12, 13]. For these reasons, this study aimed to evaluate the antibiotic susceptibility pattern, in vitro biofilm formation ability, and the prevalence of virulence genes (*esp*, *ace,* and *efaA*) among fecal normal-flora and environmental isolates of *E. faecalis* and *E. faecium*.

## Methods

### Sample collection

In this cross-sectional study, clinical and environmental samples were collected from hospital environments, healthy volunteers, and health staff of 4 educational hospitals affiliated with Mazandaran University of Medical Sciences, Sari, Iran, from October 2018 to August 2019. Participants had not taken any antibiotics for at least three weeks before sampling. The sample size was calculated according to the following formula: where n is the sample size, ${z}_{1-\frac{a}{2}}$ is the Z statistic for confidence level at 95%, p is the estimated prevalence of *E. faecalis* and *E. faecium* infections, and ${\varepsilon }^{2}$ is the precision [14].

$$n=\frac{{(z_{1-{\displaystyle\frac a2}})}^2\left[P\left(1-P\right)\right]}{\varepsilon^2}$$

### Isolation and identification of *E. faecalis* and *E. faecium*

This study strictly adhered to the principles outlined in the Declaration of Helsinki, ensuring ethical conduct throughout the research process. Approval for the study was obtained from the Iran National Committee for Ethics in Biomedical Research, with the national ethical code (consent ref number) IR.MAZUMS.REC.1398.416. Additionally, informed consent was ethically obtained from all study participants or their guardians, emphasizing our commitment to ethical standards and participant welfare. This study was approved by Biosafety committee of Mazandaran University of Medical Sciences (#1397.3490). A total of 145 clinical (stool samples, *n* = 100) and environmental samples (*n* = 45) were cultivated from four hospitals in Sari, North Iran. The samples were cultured on Slanetz and Bartley (M-Enterococcus) agar (Sigma, Germany) and blood agar (Merck, Germany) at 37°C for 24 h to isolation *Enterococcus* strains. Enterococcal species identification was done by using conventional tests (morphology of colonies, Gram staining, growth and blacken of bile-esculin agar, growth at 6.5% NaCl, 0.04% tellurite reduction, catalase test, Pyrrolidonyl arylamidase (PYR) test, arginine dehydrolase activity, motility, and some carbohydrate fermentation tests, especially arabinose [15]. The *E. faecalis* and *E. faecium* strains were confirmed by polymerase chain reaction (PCR) assay using species-specific primers for the *ddl* (D-alanine-D-alanine ligase) encoding genes.

### Antibiotic susceptibility testing

Susceptibility testing was performed using the standard Kirby Bauer disk agar diffusion method in accordance with Clinical and Laboratory Standards Institute (CLSI, 2020) guidelines. Antimicrobial agents (HiMedia, India) in this study were ampicillin (10μg), vancomycin (30μg), teicoplanin (30μg), erythromycin (15μg), tetracycline (30μg), ciprofloxacin (5μg), levofloxacin (5μg), nitrofurantoin (300μg), chloramphenicol (30μg), linezolid (300μg), gentamicin (120μg), streptomycin (300μg), and quinupristin-dalfopristin (15μg) [16]. The results of the test were interpreted according to the CLSI; M100 criteria *E. faecalis* ATCC 29212 was used as a control strain in the disk agar diffusion test.

### Biofilm formation capacity

*Enterococcus* isolates were tested for their ability to produce biofilms using a quantitative microplate assay [17]. Briefly, a 0.5 McFarland suspension of the overnight cultures of *Enterococcus* strains was prepared. To each well of 96-well micro titer plates, 180 μl of Trypticase Soy Broth (TSB; Merck, Germany) + 0.5% glucose was added along with 20 μl of 0.5 McFarland suspension of the isolates, and then incubated at 37 ˚C and 5% CO2 for 24 h. Next, the medium was discarded, and micro titer plates were gently washed three times with 300μl of sterile phosphate buffer saline (PBS) (Merck, Germany) to remove planktonic cells. Then, 150μl of 99% methanol was added to each well for 20 min to fix the biofilm biomass. Later, the methanol was removed, and the plates were left to dry in room temperature and then, 100μl of 2% crystal violet was added to each well for 20 min. Excess stains were removed from the plates using sterile distilled water and the plates were located at room temperature for 30 min. The dye bounded to the adherent cells was dissolved using 150 µL of 33% (v/v) glacial acetic acid for each well. The optical density (OD) was measured using an ELISA reader (Bio-Rad, USA) at a wavelength of 595 nm. Uninoculated TSB medium + 0.5% glucose was used as a negative control. The ability to form biofilm in these isolates was categorized based on the OD values of the strains compared to the OD cutoff (ODC) value of the control strain (*E. faecalis* ATCC 29212) into 4 separate groups: non-biofilm-formers (OD ≤ ODC), weak (ODC < OD ≤ 2 × ODC), medium (2 × ODC < OD ≤ 4 × ODC) and strong biofilm formers (4 × ODC < OD) [18].

### Polymerase chain reaction

DNAs were extracted by the alkaline lysis method following the standard protocols [19]. The distribution of *esp*, *ace*, and *efaA* genes were investigated in all *Enterococcus* isolates by PCR assay. The primer sequences used in this work are listed in Additional file 1 [20–22]. The PCR reactions contained 7.5 μl of master mix (Ampliqon, Denmark) and 0.5 μl of each primer for all genes, 100 ng of the extracted DNA for *esp* and *ddl* genes, and 200 ng DNA for *ace* and *efaA* genes. The PCR condition was as follows: an initial denaturation step at 95°C for 5 min followed by 34 cycles of denaturation at 95°C for 30 s, annealing at 54°C for *E. faecalis ddl* (45 s), 56°C for *E. faecium ddl* (45 s), 65°C for *esp* (45 s), 65°C for *efaA* and *ace* (30 s), and an extension at 72°C for *esp* and *ddl* genes (90 s) and for *ace* and *efaA* genes (60 s), with a final extension step at 72°C for 10 min (BioRad, USA). The PCR products were electrophoresed on a 1% (w/v) agarose gel (Wizbiosolutions, South Korea). Then, a UV trans-illuminator (UVITEC Gel documentation System, Cambridge, UK) was used for the documentation of the PCR products.

### Statistical analysis

Statistical analysis of results was performed with SPSS version 22 software (SPSS Chicago, IL). The Chi-square (χ^2^) and Fisher’s exact test were used for statistical analysis. A *P* value < 0.05 was used for statistical significance.

## Results

### Bacterial isolation

**Fig. 1:**
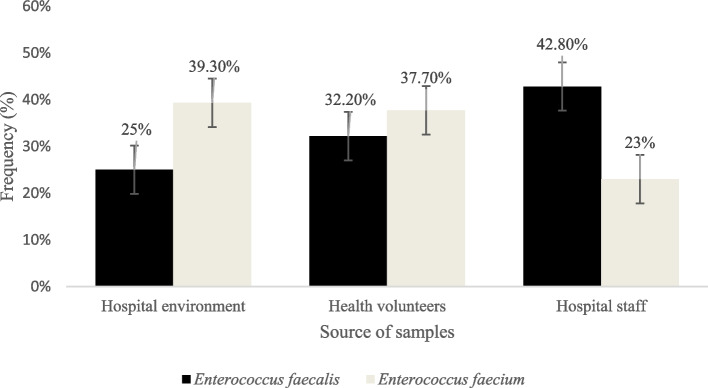
Frequency of the enterococci isolates collected from different sources

Out of 145 samples, 84 (57.9%) *E. faecalis* and 61 (42.1%) *E. faecium* were isolated. The majority of *E. faecalis* strains (36/84, 42.8%) were isolated from hospital staff, while the majority of *E. faecium* strains (24/61, 39.3%) were isolated from hospital environments (Fig. 1).

### Determination of antimicrobial susceptibility

**Fig. 2:**
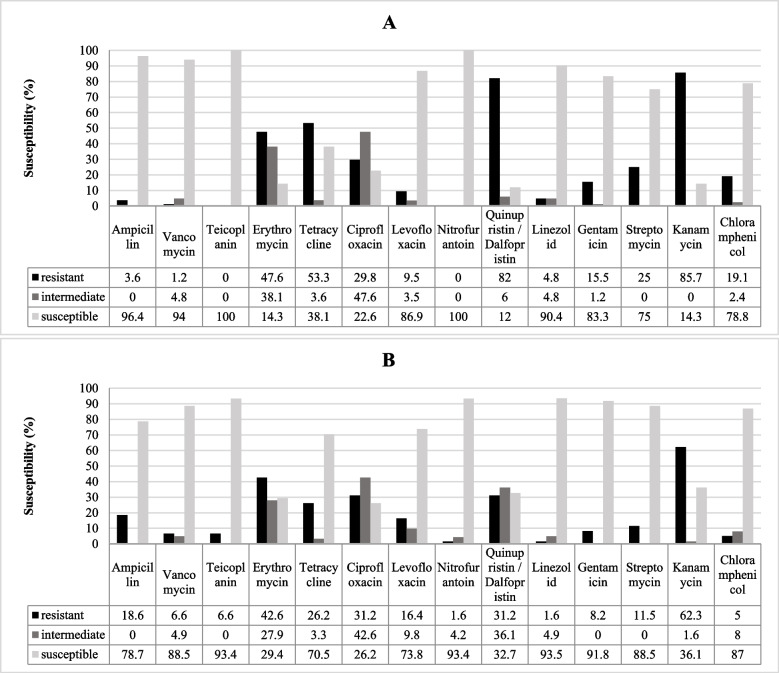
Antibiotic susceptibility of *Enterococcus* strains **A** shows the antibiotic susceptibility of *E. faecalis* and **B** shows the antibiotic susceptibility of *E. faecium*

The susceptibility profiles of tested strains are shown in Fig. 2A, B. Resistance to kanamycin (85.7%; 72/84) and quinupristin-dalfopristin (82.1%; 69/84) was high in *E. faecalis* isolates and a high prevalence of kanamycin resistance (62.3%; 38/61) was observed in the *E. faecium* isolates (Fig. 2). The antibiotic inhibition zones diameter (mm) of Enterococci isolated from hospital staffs, healthy volunteers and hospital environments are shown in Additional file 2 (Tables S2-S4).

### Distribution of virulence genes

**Fig. 3:**
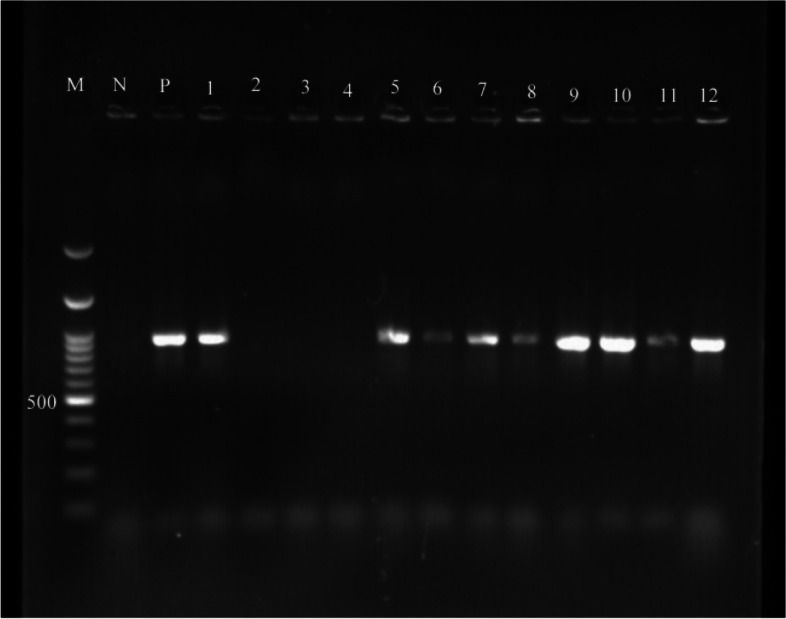
Lane M, 100–3 kb DNA size marker; Lane P, positive control; Lane N, negative control; Lane 1—12, *esp* gene positive/negative strains

**Fig. 4:**
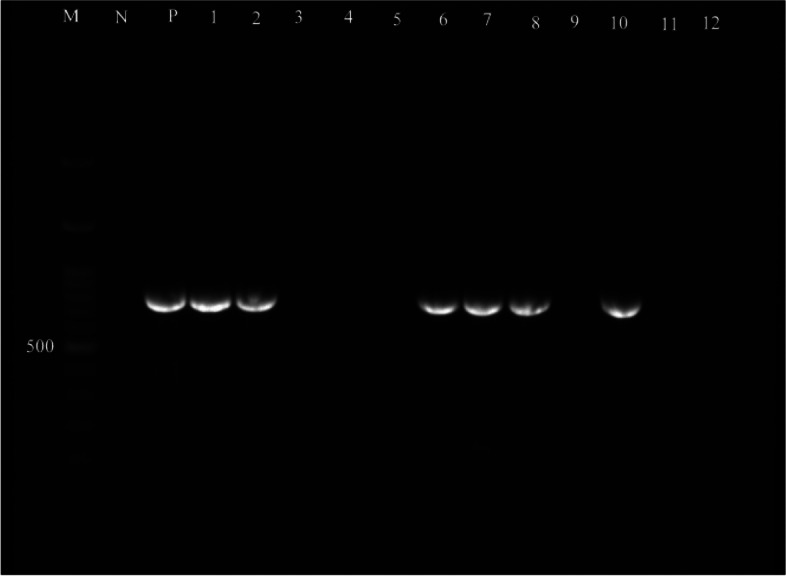
Lane M, 100–3 kb DNA size marker; Lane P, positive control; Lane N, negative control; Lane 1—12, *efa* gene positive/negative strains

**Fig. 5:**
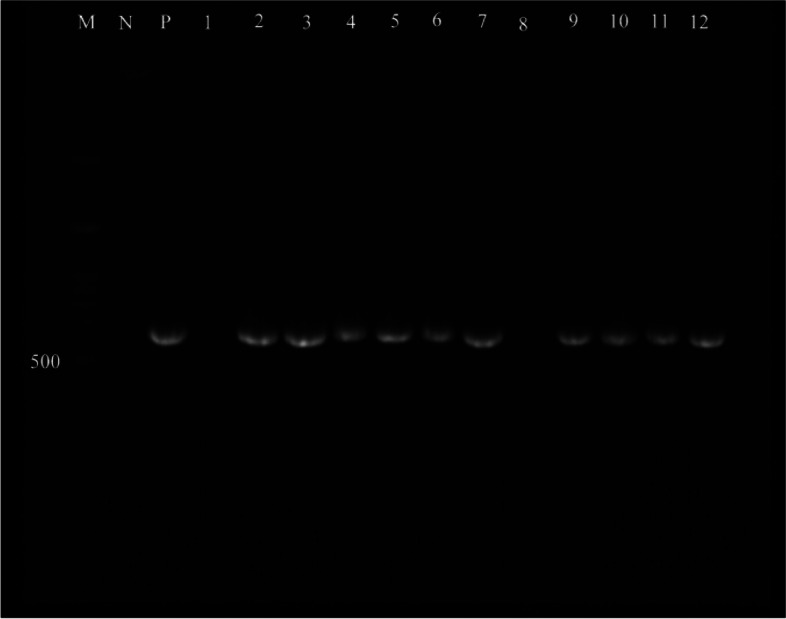
Lane M, 100–3 kb DNA size marker; Lane P, positive control; Lane N, negative control; Lane 1—12, *ace* gene positive/negative strains

All the virulence genes were screened among the Enterococcal isolates based on the occurrence of expected amplicon sizes (Figs. 3, 4 and 5). The results of PCR showed that among the *E. faecalis* isolates, 62 (73.8%) harbored the *esp* gene, 26 (30.95%) isolates had the *esp* gene, and 51 (60.71%) isolates carried the *efaA* gene. Among the *E. faecium* samples, 35 (57.37%), 3 (4.91%) and 12 (19.67%) were positive for *esp*, *ace*, and *efaA* genes, respectively (Table 1).

**Table 1:** Frequency of virulence genes among *Enterococcus* strains

Genes		*E. faecalis*	*E. faecium*	*P*-*value*
		No. (%)	No. (%)	
*esp*	+	62 (73.8)	35 (57.37)	0.038
	-	22 (26.19)	26 (42.62)	
*ace*	+	26 (30.95)	3 (4.91)	< 0.001
	-	58 (69.04)	58 (95.08)	
*efaA*	+	51 (60.71)	12 (19.67)	< 0.001
	-	33 (39.28)	49 (80.32)	

### Results of biofilm formation in *Enterococcus* strains

Based on the quantitative microplate method for biofilm formation, out of 145 enterococci strains from different sources, 99 isolates could form biofilms (Table 2). Also, there are statistically significant differences between the distributions of the *esp* gene in healthy volunteers and environmental samples of *E. faecalis* and environmental samples of *E. faecium* (*p* < 0.05). There was a statistically significant difference in the distribution of the *efaA* gene only between samples taken from healthy volunteers and environmental sources that contained *E. faecalis* (*p* < 0.05) (Table 3). The distribution of the *esp* gene among the moderate and strong phenotypes, as well as the distribution of the *efaA* gene among the moderate phenotype and *ace* gene in negative phenotype of *E. faecalis*, were statistically significant (*p* < 0.05), unlike other cases (Table 2). The results of the PCR assay indicated that there are statistically significant differences between the distributions of the *esp* gene in healthy volunteers and environmental samples of E. faecalis and environmental samples of *E. faecium* (*p* < 0.05). The distribution of the *efaA* gene showed a statistically significant difference only in samples from healthy volunteers and environmental samples of *E. faecalis* (*p* < 0.05) (Table 3). The results showed a statistically significant relationship between the presence of *esp* virulence gene and the ability of biofilm formation among *E. faecalis* isolates (*p* = 0.04). The distribution of the *esp* gene among the moderate and strong phenotypes, as well as the distribution of the *efaA* gene among the moderate phenotype and *ace* gene in negative phenotype of *E. faecalis*, were statistically significant (*p* < 0.05) (Table 4). Also correlation between antibiotic resistance pattern of the Enterococcal isolates and biofilm formation ability were assessed. The statistical analysis indicated a significant correlation between the *Enterococcus* species that form biofilms and resistance to certain antibiotics, including quinupristin/dalfopristin, streptomycin, and chloramphenicol (Table 5).

**Table 2:** Frequency of biofilm phenotypes in *Enterococcus* based on the source of samples

Group	Negative		Weak biofilm		Moderate Biofilm		Strong Biofilm		Total	
	*E. faecalis* (*n* = 16)	*E. faecium* (*n* = 30)	*E. faecalis* (*n* = 23)	*E. faecium* (*n* = 18)	*E. faecalis* (*n* = 25)	*E. faecium* (*n* = 9)	*E. faecalis* (*n* = 20)	*E. faecium* (*n* = 4)	*E. faecalis* (*n* = 84)	*E. faecium* (*n* = 61)
	No. (%)		No. (%)		No. (%)		No. (%)		No. (%)	
Hospital staff	8 (50)	7 (23.33)	10 (43.47)	2 (11.11)	9 (36)	2 (22.22)	9 (45)	3 (75)	36 (42.85)	14 (22.95)
Health volunteers	5 (31.25)	14 (46.66)	10 (43.47)	6 (33.33)	7 (28)	2 (22.22)	5 (25)	1 (25)	27 (32.14)	23 (37.70)
Hospital environment	3 (18.75)	9 (30)	3 (13.04)	10 (55.55)	9 (36)	5 (55.55)	6 (30)	0	21 (25)	24 (39.34)
*P*-*value*	0.09	0.11	0.09	0.05	0.09	0.04	0.08	0.03	0.08	0.15

**Table 3:** Frequency of virulence genes among *Enterococcus* strains based on the source of samples

Genes	Hospital staff				Health volunteers			Hospital environment			Total		
	*E. faecalis*		*E. faecium*		*E. faecalis*	*E. faecium*		*E. faecalis*	*E. faecium*		*E. faecalis*	*E. faecium*	
	No. (%)				No. (%)			No. (%)			No. (%)		
***esp***	+	20 (55.55)		3 (21.42)	21 (77.77)		9 (39.13)	21 (100)		23 (95.83)	62 (73.80)		35 (57.37)
	-	16 (44.44)		11 (78.57)	6 (22.22)		14 (60.89)	0		1 (4.16)	22 (26.19)		26 (42.62)
***P*-*value***		**0.34**		**0.58**	**0.04**		**0.48**	**0.00**		**0.01**	**0.04**		**0.31**
***ace***	+	5 (13.88)		0	15 (55.55)		1 (4.34)	6 (28.57)		2 (8.33)	26 (30.95)		3 (4.91)
	-	31 (86.11)		14 (100)	12 (44.44)		22 (95.65)	15 (71.42)		22 (91.66)	58 (69.04)		58 (95.08)
***P*-*value***		**0.84**		**SI**	**0.34**		**1.52**	**0.51**		**0.98**	**0.44**		**1.52**
***efaA***	+	7 (19.44)		0	24 (88.88)		5 (21.73)	20 (95.23)		7 (29.16)	51 (60.71)		12 (19.67)
	-	29 (80.55)		14 (100)	3 (11.11)		18 (78.26)	1 (4.76)		17 (70.83)	33 (39.28)		49 (80.32)
***P*-*value***		**0.54**		**SI**^a^	**0.03**		**0.58**	**0.01**		**0.46**	**0.29**		**0.54**

^a^*Abbreviation: SI* Statistically Incalculable

**Table 4:** Frequency of biofilm phenotypes in *Enterococcus* isolates based on the distribution of virulence genes

Genes		Negative		Weak biofilm		Moderate Biofilm		Strong Biofilm		Total of biofilm positive	
		*E. faecalis*	*E. faecium*	*E. faecalis*	*E. faecium*	*E. faecalis*	*E. faecium*	*E. faecalis*	*E. faecium*	*E. faecalis*	*E. faecium*
		No. (%)		No. (%)		No. (%)		No. (%)		No. (%)	
***esp***	+	12 (75)	16 (53.33)	15 (65.21)	12 (66.66)	20 (80)	5 (55.55)	15 (75)	2 (50)	50 (73.52)	19 (61.29)
	-	4 (25)	14 (46.66)	8 (34.78)	6 (33.33)	5 (20)	4 (44.44)	5 (25)	2 (50)	18 (26.47)	12 (38.70)
***P*-*value***		**1.11**	**0.20**	**0.15**	**0.11**	**0.03**	**0.18**	**0.04**	**0.25**	**0.04**	**0.18**
***ace***	+	3 (18.75)	0	8 (34.78)	3 (16.66)	8 (32)	0	7 (35)	0	23 (33.82)	3 (9.67)
	-	13 (81.25)	30 (100)	15 (65.21)	15 (83.33)	17 (68)	9 (100)	13 (65)	4 (100)	45 (66.17)	28 (90.32)
***P*-*value***		**0.03**	**SI**	**0.94**	**1.21**	**0.98**	**SI**	**0.46**	**SI**	**0.48**	**1.44**
***efaA***	+	8 (50)	6 (20)	15 (65.21)	3 (16.66)	17 (68)	3 (33.33)	11 (55)	0	43 (63.23)	6 (19.35)
	-	8 (50)	24 (80)	8 (34.78)	15 (83.33)	8 (32)	6 (66.66)	9 (45)	4 (100)	25 (36.76)	25 (80.64)
***P*-*value***		**0.25**	**0.71**	**0.15**	**1.21**	**0.04**	**0.49**	**0.19**	**SI**^a^	**0.16**	**1.10**

^a^*Abbreviation: SI* Statistically Incalculabl

**Table 5:** Correlation between antibiotic resistance pattern of the Enterococcal isolates and biofilm formation ability

Antibiotics	Antimicrobial resistance pattern	No. (%) of isolates with biofilm production ability				Total	*P*- *value*
		Negative	Weak	Moderate	Strong		
Ampicillin	Resistant	9 (56.2)	3 (18.7)	3 (18.7)	1 (6.2)	16	0.150
	Intermediate Resistant	-	-	-	-	-	
	Susceptible	37 (28.6)	38 (29.4)	31 (24.0)	23 (17.8)	129	
Vancomycin	Resistant	3 (60)	2 (40)	0	0	5	0.277
	Intermediate Resistant	2 (28.5)	4 (57.1)	1 (14.2)	0	7	
	Susceptible	41 (30.8)	35 (26.3)	33 (24.8)	24 (18.0)	133	
Teicoplanin	Resistant	2 (50)	2 (50)	0	0	4	0.429
	Intermediate Resistant	-	-	-	-	-	
	Susceptible	44 (31.2)	39 (27.6)	34 (24.1)	24 (17.0)	141	
Erythromycin	Resistant	19 (28.7)	18 (27.2)	15 (22.7)	14 (21.2)	66	0.899
	Intermediate Resistant	16 (32.6)	15 (30.6)	12 (24.4)	6 (12.2)	49	
	Susceptible	11 (36.6)	8 (26.6)	7 (23.3)	4 (13.3)	30	
Tetracycline	Resistant	18 (27.6)	16 (24.6)	14 (21.5)	17 (26.1)	65	0.180
	Intermediate Resistant	1 (20)	2 (40)	1 (20)	1 (20)	5	
	Susceptible	27 (36)	23 (30.6)	19 (25.3)	6 (8)	75	
Ciprofloxacin	Resistant	17 (38.6)	10 (22.7)	11 (25)	6 (13.6)	44	0.885
	Intermediate Resistant	20 (30.3)	20 (30.3)	15 (22.7)	11 (16.6)	66	
	Susceptible	9 (25.7)	11 (31.4)	8 (22.8)	7 (20)	35	
Levofloxacin	Resistant	5 (27.7)	6 (33.3)	4 (22.2)	3 (16.6)	18	0.144
	Intermediate Resistant	7 (70)	3 (30)	0	0	10	
	Susceptible	34 (29.0)	32 (27.3)	30 (25.6)	21 (17.9)	117	
Nitrofurantoin	Resistant	1 (100)	0	0	0	1	0.182
	Intermediate Resistant	3 (100)	0	0	0	3	
	Susceptible	42 (29.7)	41 (29.0)	34 (24.1)	24 (17.0)	141	
Quinupristin/dalfopristin	Resistant	23 (26.1)	24 (27.2)	23 (26.1)	18 (20.4)	88	0.024
	Intermediate Resistant	16 (59.2)	5 (18.5)	5 (18.5)	1 (3.7)	27	
	Susceptible	7 (23.3)	12 (40)	6 (20)	5 (16.6)	30	
Linezolid	Resistant	0	4 (80)	0	1 (20)	5	0.156
	Intermediate Resistant	2 (28.5)	1 (14.2)	3 (42.8)	1 (14.2)	7	
	Susceptible	44 (33.0)	36 (27.0)	31 (23.3)	22 (16.5)	133	
Gentamicin	Resistant	6 (33.3)	4 (22.2)	3 (16.6)	5 (27.7)	18	0.486
	Intermediate Resistant	0	0	1 (100)	0	1	
	Susceptible	40 (31.7)	37 (29.3)	30 (23.8)	19 (15.0)	126	
Streptomycin	Resistant	9 (32.1)	5 (17.8)	4 (14.2)	10 (35.7)	28	0.016
	Intermediate Resistant	-	-	-	-	-	
	Susceptible	37 (31.6)	36 (30.7)	30 (25.6)	14 (11.9)	117	
Kanamycin	Resistant	37 (33.6)	27 (24.5)	27 (24.5)	19 (17.2)	110	0.547
	Intermediate Resistant	0	1 (100)	0	0	1	
	Susceptible	9 (26.4)	13 (38.2)	7 (20.5)	5 (14.7)	34	
Chloramphenicol	Resistant	4 (21.0)	4 (21.0)	3 (15.7)	8 (42.1)	19	0.026
	Intermediate Resistant	2 (28.5)	4 (57.1)	0	1 (14.2)	7	
	Susceptible	40 (33.6)	33 (27.7)	31 (26.0)	15 (12.6)	119	

## Discussion

A number of severe and life-threatening diseases can be caused by enterococci [23]. *E. faecalis* and *E. faecium* are the most commonly detected species of enterococci in human clinical samples [24]. Among the 145 *Enterococcus* isolates in this study, 57.9% were *E. faecalis* and 42.1% were *E. faecium*. In several other studies, *E. faecalis* was the predominant strain. The incidence of *E. faecalis* as a predominani enterococci strains has been reported to vary from 70% (in Tehran, Iran), 69% (in Zanjan, Iran), and 41.99% (in China) [25–27]. The difference in the prevalence of *E. faecalis* and *E. faecium* can be due to differences in the type of samples, methods of detection, or geographical location. Enterococci are innately resistant to antibiotics, but can acquire resistance genes and new mutations from other bacteria as well [28]. Several studies in Iran have reported high rates of antibiotic resistance among *Enterococcus* strains [29, 30]. A high level of kanamycin resistance was detected in 85.7% and 62.3% of *E. faecium* and *E. faecalis* isolates, respectively. Although intrinsic resistance mechanisms may result in low levels of aminoglycoside resistance, acquiring mobile genetic elements usually leads to high levels of aminoglycoside resistance in these isolates [31]. Additionally, ampicillin resistance in *E. faecium* isolates of the present study was considerable, similar to a previous study in Kenya [32]. On the other hand, several virulence genes (*efaA*, *asa1*, *ebpA*, *esp*, and *ace*) have been identified as effective genes for biofilm formation in *Enterococci* [33]. In our study, the prevalence of *ace, esp,* and* efaA* genes among *E. faecalis* isolates, were 74%, 31%, and 31.1%, respectively, while 57%, 5%, and 31.1% of *E. faecium* isolates contained these genes, respectively. A number of virulence genes were found in our study to be consistent with those found in previous studies conducted on food, animal, and medical isolates [22, 34, 35]. Among these two common enterococci species, the prevalence of the *esp* gene varies from one country to the next [12, 36]. However, enterococcal surface protein (Esp) is one of the most important factors in colonization and persistence of *E. faecalis* in human urinary tract infections and its biofilm formation [12, 37]. The *esp* gene has been detected in clinical and environmental samples in the past [22, 39], but they are more commonly adopted in clinical isolates [39]. There is a wide variation in the distribution of the *esp* gene in enterococci even within the same geographic region [37]. Lenz et al. report that *efaA* plays a significant role in response to bile salt stress in *E. faecalis* strains [38]. Biofilm formation in enterococci is directly affected by *esp*, *efaA*, and *ace* genes, based on the phenotypic results and the presense of these selected genes. According to the findings of the study, *esp* and *efaA* genes were more frequently found among *E. faecalis* strains with moderate and strong biofilm forming capability. Several studies have also reported similar findings [36]. It has been demonstrated that the *esp* gene plays an important role in the formation of biofilm [39].

One limitation inherent in these studies is the potential impact of the surface, culture medium, and duration chosen for biofilm formation on the resulting strength of biofilm production. In future investigations, it is imperative to thoroughly explore and address this limitation to enhance our overall comprehension of the factors influencing biofilm formation.

## Conclusion

The results of this study revealed a notable increase in resistance levels to kanamycin, tetracycline, and streptogramin. Our interpretation suggests a potential correlation between this elevated resistance and the intensive use of tetracycline and kanamycin for various purposes within the studied region. The widespread application of these antibiotics, whether in medical, agricultural, or other contexts, may contribute to the emergence and persistence of resistance patterns observed in this study. This correlation underscores the need for a comprehensive understanding of antibiotic usage practices and their impact on antibiotic resistance within the specific geographic context of our study. Also, the increase in resistance to streptogramin showed the importance of MLS_B_ (macrolide-lincosamide-streptogramin B) resistance phenotypes in enterococci. Eventually, we showed that the presense of *esp*, *ace*, and *efaA* genes in *E. faecalis* was higher than in *E. faecium*, which could be due to the high expression of these genes in *E. faecalis*. The control of enterococcal infections in hospitals may be affected by the presence of the *esp*, *efaA*, and *ace* genes in *E. faecium* and *E. faecalis* isolates, which would maintain their establishment and growth in hospital settings.

### Suggestions

The presence of other genes related to the biofilm production should be investigated. Study on clinical isolates collected from hospitalized patients infected with *enterococcus* isolates should be performed. Also, in order to achieve better results, the mulecular typing techniques, such as RAPD-PCR and PFGE, are necessary to assess the sources and/or diversity of the strains.

### Supplementary Information

**Additional file 1: Table S1.** List of primers used in PCR test for detection of *esp*, *ace*, and *efaA* genes.

**Additional file 2: Table S2.** The antibiotic inhibition zones diameter (mm) of Enterococci isolated from hospital staffs. **Table S3.** The antibiotic inhibition zones diameter (mm) of Enterococci isolated from healthy volunteers. **Table S4.** The antibiotic inhibition zones diameter (mm) of Enterococci isolated from hospital environments.

## Data Availability

Data generated or analyzed during this study are included in this published article.
