# Supplementary material for: Antimicrobial resistance patterns, virulence genes, and biofilm formation in enterococci strains collected from different sources
Source: BMC Infect Dis. 2024 Mar 4;24:274. doi: 10.1186/s12879-024-09117-2 (PMC10910731; doi:10.1186/s12879-024-09117-2)
Supplement: Supplementary file 1 — Additional file 1: Table S1. List of primers used in PCR test for detection of esp, ace, and efaA genes. [file 12879_2024_9117_MOESM1_ESM.docx]

**Additional file 1: Table S1:** List of primers used in PCR test for detection of *esp*, *ace*, and *efaA* genes

| Genes | Sequence (5΄–3΄) | Product size (bp) | Reference |
| --- | --- | --- | --- |
| *ddl-F* | ATCAAGTACAGTTAGTCT | 941 | ([20](#_ENREF_20)) |
| *ddl-R* | ACGATTCAAAGCTAACTG |  |  |
| *ace –F* | GAATGACCGAGAACGATGGC | 615 | ([21](#_ENREF_21)) |
| *ace –R* | CTTGATGTTGGCCTGCTTCC |  |  |
| *efaA-F* | GACAGACCCTCACGAATATG | 706 | ([21](#_ENREF_21)) |
| *efaA-R* | CCAGTTCATCATGCTGTAGTA |  |  |
| *esp-F* | TTGCTAATGCTAGTCCACGACC | 955 | ([22](#_ENREF_22)) |
| *esp-R* | GCGTCAACACTTGCATTGCCGAA |  |  |
