# Supplementary material for: Antimicrobial resistance patterns, virulence genes, and biofilm formation in enterococci strains collected from different sources
Source: BMC Infect Dis. 2024 Mar 4;24:274. doi: 10.1186/s12879-024-09117-2 (PMC10910731; doi:10.1186/s12879-024-09117-2)
Supplement: Supplementary file 2 — Additional file 2: Table S2. The antibiotic inhibition zones diameter (mm) of Enterococci isolated from hospital staffs. Table S3. The antibiotic inhibition zones diameter (mm) of Enterococci isolated from healthy volunteers. Table S4. The antibiotic inhibition zones diameter (mm) of Enterococci isolated from hospital environments. [file 12879_2024_9117_MOESM2_ESM.docx]

Additional file 2: Table S2: The antibiotic inhibition zones diameter (mm) of Enterococci isolated from hospital staffs

| Number | AMP | VAN | TPN | ERY | TET | CIP | LEV | NIT | QD | LIN | GEN | STR | KAN | CLR |
| --- | --- | --- | --- | --- | --- | --- | --- | --- | --- | --- | --- | --- | --- | --- |
| 1 | 28 | 20 | 18 | 0 | 0 | 20 | 23 | 27 | 20 | 25 | 19 | 17 | 0 | 25 |
| 2 | 30 | 20 | 25 | 0 | 0 | 0 | 0 | 28 | 0 | 0 | 0 | 0 | 0 | 0 |
| 3 | 30 | 20 | 23 | 0 | 33 | 23 | 24 | 27 | 10 | 30 | 20 | 15 | 0 | 23 |
| 4 | 25 | 25 | 17 | 17 | 30 | 10 | 17 | 25 | 26 | 30 | 20 | 20 | 20 | 24 |
| 5 | 28 | 20 | 23 | 0 | 33 | 24 | 23 | 28 | 10 | 28 | 15 | 17 | 0 | 25 |
| 6 | 28 | 18 | 15 | 26 | 30 | 12 | 30 | 20 | 15 | 26 | 20 | 25 | 25 | 24 |
| 7 | 28 | 28 | 15 | 15 | 30 | 20 | 20 | 25 | 10 | 25 | 15 | 18 | 0 | 23 |
| 8 | 20 | 20 | 18 | 12 | 28 | 15 | 15 | 20 | 15 | 21 | 20 | 20 | 21 | 20 |
| 9 | 30 | 20 | 20 | 23 | 30 | 18 | 23 | 30 | 14 | 28 | 20 | 15 | 0 | 23 |
| 10 | 26 | 20 | 20 | 23 | 30 | 20 | 20 | 25 | 25 | 15 | 18 | 18 | 0 | 25 |
| 11 | 26 | 24 | 19 | 17 | 28 | 19 | 18 | 25 | 24 | 26 | 19 | 22 | 20 | 24 |
| 12 | 25 | 22 | 20 | 17 | 0 | 15 | 15 | 20 | 18 | 30 | 20 | 13 | 9 | 25 |
| 13 | 26 | 19 | 19 | 0 | 0 | 24 | 23 | 25 | 11 | 31 | 21 | 0 | 21 | 12 |
| 14 | 25 | 22 | 22 | 24 | 28 | 20 | 20 | 25 | 16 | 28 | 18 | 17 | 0 | 23 |
| 15 | 30 | 20 | 20 | 0 | 12 | 20 | 23 | 28 | 13 | 30 | 18 | 0 | 0 | 0 |
| 16 | 23 | 20 | 19 | 0 | 12 | 23 | 21 | 24 | 10 | 28 | 19 | 0 | 0 | 12 |
| 17 | 30 | 20 | 19 | 0 | 15 | 25 | 20 | 23 | 11 | 27 | 20 | 0 | 0 | 0 |
| 18 | 28 | 22 | 20 | 25 | 30 | 15 | 19 | 23 | 25 | 28 | 26 | 23 | 26 | 25 |
| 19 | 27 | 21 | 23 | 28 | 30 | 25 | 23 | 20 | 19 | 23 | 18 | 15 | 0 | 21 |
| 20 | 25 | 19 | 20 | 15 | 0 | 19 | 20 | 26 | 12 | 28 | 17 | 15 | 0 | 25 |
| 21 | 28 | 20 | 20 | 25 | 13 | 20 | 20 | 27 | 12 | 21 | 18 | 18 | 0 | 24 |
| 22 | 36 | 28 | 20 | 20 | 37 | 12 | 17 | 20 | 28 | 28 | 26 | 26 | 21 | 15 |
| 23 | 25 | 20 | 20 | 0 | 25 | 17 | 28 | 20 | 18 | 25 | 18 | 15 | 0 | 21 |
| 24 | 30 | 20 | 20 | 18 | 0 | 20 | 20 | 24 | 12 | 25 | 17 | 0 | 0 | 24 |
| 25 | 28 | 20 | 20 | 18 | 12 | 19 | 20 | 24 | 17 | 29 | 21 | 20 | 0 | 25 |
| 26 | 25 | 20 | 20 | 0 | 0 | 20 | 20 | 25 | 0 | 26 | 0 | 15 | 0 | 22 |
| 27 | 25 | 20 | 20 | 20 | 27 | 15 | 17 | 25 | 5 | 30 | 15 | 15 | 0 | 25 |
| 28 | 25 | 18 | 20 | 0 | 0 | 20 | 24 | 25 | 10 | 28 | 0 | 15 | 0 | 25 |
| 29 | 30 | 20 | 20 | 23 | 27 | 18 | 20 | 22 | 12 | 27 | 26 | 15 | 0 | 25 |
| 30 | 25 | 20 | 19 | 23 | 30 | 20 | 20 | 25 | 12 | 25 | 17 | 12 | 0 | 25 |
| 31 | 28 | 21 | 26 | 16 | 30 | 18 | 23 | 27 | 0 | 28 | 21 | 19 | 0 | 26 |
| 32 | 27 | 22 | 24 | 25 | 31 | 19 | 24 | 28 | 0 | 27 | 22 | 20 | 0 | 25 |
| 33 | 26 | 22 | 25 | 16 | 30 | 18 | 25 | 27 | 0 | 28 | 21 | 23 | 0 | 26 |
| 34 | 28 | 23 | 26 | 8 | 10 | 19 | 26 | 28 | 15 | 27 | 23 | 0 | 0 | 25 |
| 35 | 27 | 25 | 25 | 26 | 10 | 18 | 10 | 27 | 0 | 28 | 25 | 0 | 0 | 26 |
| 36 | 15 | 25 | 25 | 0 | 0 | 0 | 15 | 27 | 0 | 28 | 0 | 0 | 0 | 27 |
| 37 | 28 | 20 | 26 | 0 | 0 | 0 | 15 | 28 | 15 | 27 | 25 | 23 | 0 | 26 |
| 38 | 27 | 20 | 24 | 16 | 0 | 0 | 25 | 27 | 25 | 27 | 22 | 22 | 0 | 27 |
| 39 | 27 | 20 | 25 | 24 | 30 | 26 | 24 | 27 | 15 | 26 | 25 | 23 | 0 | 25 |
| 40 | 26 | 21 | 24 | 16 | 0 | 0 | 25 | 27 | 0 | 26 | 22 | 22 | 0 | 26 |
| 41 | 25 | 20 | 26 | 16 | 0 | 0 | 24 | 26 | 0 | 25 | 22 | 23 | 0 | 25 |
| 42 | 26 | 0 | 24 | 25 | 30 | 18 | 25 | 25 | 0 | 26 | 0 | 22 | 0 | 24 |
| 43 | 25 | 21 | 25 | 0 | 0 | 0 | 24 | 26 | 0 | 25 | 0 | 23 | 0 | 25 |
| 44 | 27 | 20 | 25 | 26 | 0 | 0 | 25 | 25 | 0 | 24 | 25 | 22 | 0 | 24 |
| 45 | 22 | 18 | 17 | 18 | 25 | 17 | 20 | 20 | 11 | 23 | 16 | 15 | 0 | 20 |
| 46 | 20 | 19 | 21 | 18 | 30 | 22 | 20 | 25 | 14 | 28 | 17 | 15 | 0 | 22 |
| 47 | 20 | 19 | 20 | 17 | 35 | 20 | 20 | 27 | 20 | 30 | 23 | 15 | 0 | 25 |
| 48 | 27 | 20 | 20 | 20 | 32 | 24 | 24 | 27 | 11 | 28 | 17 | 16 | 0 | 25 |
| 49 | 23 | 20 | 18 | 14 | 0 | 19 | 18 | 26 | 23 | 30 | 18 | 0 | 0 | 12 |
| 50 | 27 | 20 | 20 | 23 | 30 | 13 | 21 | 24 | 13 | 26 | 20 | 20 | 21 | 25 |

Abbreviations: AMP: Ampicillin; VAN: Vancomycin; TPN: Teicoplanin; ERY: Erythromycin; TET: Tetracycline; CIP: Ciprofloxacin; LEV: Levofloxacin; NIT: Nitrofurantoin; QD: Quinupristin/Dalfopristin; LIN: Linezolid; GEN: Gentamicin; STR: Streptomycin; KAN: Kanamycin; CLR: Chloramphenicol.

Additional file 2: Table S3: The antibiotic inhibition zones diameter (mm) of Enterococci isolated from healthy volunteers

| Number | AMP | VAN | TPN | ERY | TET | CIP | LEV | NIT | QD | LIN | GEN | STR | KAN | CLR |
| --- | --- | --- | --- | --- | --- | --- | --- | --- | --- | --- | --- | --- | --- | --- |
| 1 | 20 | 17 | 19 | 14 | 29 | 19 | 20 | 22 | 17 | 27 | 23 | 20 | 0 | 26 |
| 2 | 28 | 20 | 21 | 0 | 11 | 23 | 22 | 27 | 10 | 30 | 18 | 17 | 0 | 23 |
| 3 | 30 | 20 | 20 | 20 | 29 | 25 | 24 | 25 | 10 | 23 | 18 | 15 | 0 | 22 |
| 4 | 25 | 20 | 18 | 12 | 30 | 23 | 22 | 23 | 16 | 27 | 20 | 20 | 0 | 23 |
| 5 | 30 | 18 | 18 | 20 | 12 | 20 | 20 | 25 | 15 | 28 | 20 | 18 | 0 | 24 |
| 6 | 22 | 15 | 17 | 14 | 27 | 16 | 19 | 20 | 13 | 25 | 18 | 18 | 20 | 20 |
| 7 | 25 | 17 | 18 | 17 | 0 | 16 | 18 | 22 | 13 | 20 | 16 | 14 | 22 | 23 |
| 8 | 28 | 20 | 20 | 0 | 0 | 0 | 0 | 28 | 8 | 26 | 7 | 0 | 0 | 21 |
| 9 | 27 | 20 | 21 | 0 | 18 | 25 | 25 | 25 | 15 | 26 | 0 | 0 | 0 | 0 |
| 10 | 27 | 28 | 19 | 14 | 11 | 25 | 23 | 25 | 14 | 30 | 0 | 0 | 0 | 20 |
| 11 | 28 | 15 | 17 | 12 | 25 | 17 | 17 | 20 | 14 | 27 | 16 | 12 | 0 | 24 |
| 12 | 28 | 17 | 17 | 14 | 15 | 12 | 16 | 24 | 13 | 25 | 15 | 15 | 20 | 17 |
| 13 | 23 | 27 | 18 | 19 | 13 | 16 | 20 | 23 | 14 | 27 | 21 | 0 | 0 | 22 |
| 14 | 24 | 20 | 19 | 15 | 0 | 12 | 17 | 18 | 15 | 27 | 20 | 18 | 0 | 24 |
| 15 | 16 | 20 | 18 | 0 | 30 | 28 | 20 | 25 | 18 | 30 | 20 | 30 | 30 | 25 |
| 16 | 25 | 16 | 23 | 23 | 24 | 18 | 20 | 25 | 21 | 23 | 15 | 29 | 21 | 23 |
| 17 | 24 | 19 | 19 | 15 | 30 | 17 | 12 | 18 | 18 | 27 | 20 | 20 | 0 | 21 |
| 18 | 27 | 18 | 19 | 17 | 0 | 18 | 20 | 25 | 20 | 24 | 15 | 15 | 0 | 23 |
| 19 | 0 | 19 | 18 | 20 | 23 | 25 | 28 | 21 | 16 | 28 | 25 | 23 | 20 | 26 |
| 20 | 25 | 17 | 20 | 13 | 25 | 18 | 15 | 28 | 10 | 24 | 15 | 15 | 0 | 22 |
| 21 | 26 | 22 | 18 | 0 | 25 | 18 | 20 | 18 | 18 | 28 | 17 | 15 | 0 | 23 |
| 22 | 30 | 18 | 17 | 15 | 0 | 17 | 20 | 21 | 15 | 25 | 17 | 15 | 0 | 20 |
| 23 | 28 | 21 | 18 | 0 | 28 | 18 | 20 | 20 | 15 | 27 | 20 | 23 | 0 | 25 |
| 24 | 25 | 19 | 17 | 17 | 0 | 15 | 0 | 20 | 15 | 30 | 20 | 17 | 0 | 8 |
| 25 | 25 | 18 | 20 | 30 | 0 | 20 | 20 | 30 | 18 | 23 | 20 | 18 | 0 | 23 |
| 26 | 23 | 16 | 17 | 18 | 18 | 15 | 18 | 19 | 12 | 21 | 18 | 17 | 0 | 17 |
| 27 | 26 | 17 | 18 | 15 | 14 | 15 | 17 | 21 | 15 | 21 | 16 | 14 | 0 | 20 |
| 28 | 25 | 19 | 17 | 0 | 0 | 18 | 20 | 21 | 0 | 25 | 17 | 15 | 0 | 16 |
| 29 | 30 | 18 | 28 | 15 | 20 | 17 | 20 | 23 | 15 | 25 | 18 | 15 | 0 | 24 |
| 30 | 30 | 20 | 20 | 19 | 31 | 22 | 19 | 24 | 16 | 27 | 21 | 20 | 0 | 23 |
| 31 | 21 | 17 | 17 | 0 | 0 | 16 | 20 | 18 | 15 | 23 | 15 | 23 | 0 | 20 |
| 32 | 25 | 23 | 20 | 0 | 30 | 20 | 18 | 20 | 18 | 26 | 20 | 24 | 0 | 22 |
| 33 | 24 | 20 | 19 | 0 | 0 | 26 | 23 | 23 | 12 | 25 | 0 | 22 | 0 | 23 |
| 34 | 28 | 23 | 18 | 13 | 30 | 18 | 20 | 24 | 18 | 25 | 20 | 20 | 0 | 23 |
| 35 | 28 | 22 | 18 | 0 | 25 | 17 | 19 | 18 | 15 | 23 | 20 | 20 | 0 | 21 |
| 36 | 28 | 23 | 17 | 0 | 28 | 19 | 20 | 22 | 15 | 24 | 20 | 20 | 0 | 22 |
| 37 | 23 | 20 | 18 | 21 | 27 | 13 | 16 | 16 | 23 | 23 | 20 | 18 | 0 | 22 |
| 38 | 26 | 18 | 18 | 17 | 25 | 18 | 19 | 22 | 11 | 23 | 15 | 13 | 0 | 18 |
| 39 | 11 | 18 | 17 | 25 | 21 | 18 | 20 | 18 | 15 | 25 | 25 | 23 | 21 | 23 |
| 40 | 21 | 15 | 16 | 0 | 0 | 19 | 18 | 19 | 11 | 21 | 15 | 0 | 0 | 0 |
| 41 | 0 | 18 | 23 | 28 | 24 | 19 | 23 | 20 | 17 | 28 | 21 | 23 | 21 | 17 |
| 42 | 30 | 19 | 18 | 33 | 33 | 26 | 25 | 25 | 22 | 30 | 21 | 30 | 24 | 23 |
| 43 | 26 | 20 | 18 | 21 | 27 | 0 | 12 | 22 | 15 | 25 | 19 | 19 | 21 | 21 |
| 44 | 23 | 17 | 15 | 15 | 26 | 19 | 18 | 21 | 17 | 21 | 16 | 13 | 0 | 20 |
| 45 | 21 | 21 | 18 | 12 | 28 | 20 | 20 | 20 | 18 | 23 | 19 | 16 | 0 | 21 |
| 46 | 24 | 16 | 18 | 0 | 0 | 15 | 17 | 24 | 0 | 20 | 17 | 14 | 0 | 0 |
| 47 | 24 | 15 | 16 | 0 | 0 | 16 | 15 | 21 | 11 | 20 | 16 | 0 | 0 | 0 |
| 48 | 24 | 17 | 17 | 15 | 26 | 11 | 12 | 17 | 12 | 28 | 20 | 15 | 0 | 20 |
| 49 | 24 | 20 | 17 | 14 | 23 | 15 | 15 | 16 | 21 | 28 | 20 | 19 | 0 | 23 |
| 50 | 20 | 21 | 17 | 0 | 25 | 14 | 15 | 13 | 16 | 25 | 18 | 16 | 0 | 20 |

Abbreviations: AMP: Ampicillin; VAN: Vancomycin; TPN: Teicoplanin; ERY: Erythromycin; TET: Tetracycline; CIP: Ciprofloxacin; LEV: Levofloxacin; NIT: Nitrofurantoin; QD: Quinupristin/Dalfopristin; LIN: Linezolid; GEN: Gentamicin; STR: Streptomycin; KAN: Kanamycin; CLR: Chloramphenicol.

Additional file 2: Table S4: The antibiotic inhibition zones diameter (mm) of Enterococci isolated from hospital environments

| Number | AMP | VAN | TPN | ERY | TET | CIP | LEV | NIT | QD | LIN | GEN | STR | KAN | CLR |
| --- | --- | --- | --- | --- | --- | --- | --- | --- | --- | --- | --- | --- | --- | --- |
| 1 | 25 | 18 | 17 | 0 | 0 | 20 | 20 | 22 | 11 | 27 | 0 | 0 | 0 | 26 |
| 2 | 28 | 19 | 20 | 20 | 16 | 21 | 21 | 24 | 11 | 25 | 17 | 18 | 0 | 19 |
| 3 | 28 | 20 | 21 | 28 | 25 | 22 | 22 | 25 | 25 | 26 | 19 | 20 | 21 | 20 |
| 4 | 30 | 20 | 20 | 0 | 0 | 0 | 0 | 28 | 10 | 27 | 14 | 0 | 0 | 0 |
| 5 | 25 | 17 | 20 | 15 | 0 | 20 | 20 | 20 | 13 | 24 | 17 | 0 | 0 | 0 |
| 6 | 28 | 18 | 22 | 28 | 24 | 26 | 24 | 25 | 23 | 28 | 20 | 23 | 25 | 24 |
| 7 | 27 | 19 | 23 | 30 | 30 | 27 | 26 | 22 | 22 | 30 | 21 | 30 | 24 | 23 |
| 8 | 26 | 20 | 21 | 26 | 31 | 28 | 24 | 21 | 25 | 25 | 22 | 21 | 23 | 26 |
| 9 | 25 | 20 | 20 | 0 | 0 | 0 | 0 | 24 | 0 | 25 | 0 | 0 | 0 | 16 |
| 10 | 0 | 0 | 0 | 0 | 0 | 0 | 0 | 23 | 13 | 27 | 0 | 20 | 0 | 21 |
| 11 | 0 | 0 | 0 | 0 | 0 | 0 | 0 | 30 | 12 | 33 | 0 | 0 | 0 | 27 |
| 12 | 33 | 19 | 20 | 23 | 30 | 27 | 18 | 21 | 18 | 25 | 12 | 23 | 21 | 21 |
| 13 | 0 | 0 | 20 | 25 | 16 | 11 | 0 | 27 | 20 | 25 | 20 | 12 | 0 | 40 |
| 14 | 25 | 18 | 20 | 12 | 0 | 15 | 17 | 20 | 10 | 30 | 20 | 0 | 0 | 25 |
| 15 | 27 | 20 | 19 | 0 | 0 | 19 | 23 | 21 | 11 | 25 | 12 | 17 | 0 | 18 |
| 16 | 28 | 18 | 18 | 32 | 30 | 22 | 33 | 22 | 27 | 25 | 18 | 17 | 22 | 30 |
| 17 | 30 | 24 | 22 | 23 | 30 | 20 | 20 | 27 | 17 | 28 | 20 | 18 | 20 | 24 |
| 18 | 25 | 25 | 20 | 13 | 33 | 15 | 18 | 28 | 17 | 34 | 20 | 20 | 0 | 30 |
| 19 | 30 | 21 | 22 | 25 | 14 | 23 | 23 | 30 | 24 | 30 | 19 | 17 | 0 | 25 |
| 20 | 0 | 19 | 17 | 0 | 0 | 0 | 12 | 20 | 20 | 33 | 16 | 30 | 0 | 23 |
| 21 | 21 | 21 | 21 | 5 | 14 | 21 | 25 | 28 | 18 | 27 | 20 | 16 | 0 | 27 |
| 22 | 21 | 21 | 20 | 0 | 0 | 23 | 25 | 27 | 12 | 21 | 20 | 16 | 0 | 28 |
| 23 | 26 | 21 | 20 | 19 | 25 | 21 | 21 | 27 | 26 | 29 | 22 | 19 | 21 | 26 |
| 24 | 25 | 23 | 22 | 21 | 25 | 22 | 24 | 26 | 25 | 28 | 21 | 23 | 21 | 28 |
| 25 | 0 | 0 | 0 | 0 | 0 | 0 | 0 | 16 | 12 | 34 | 0 | 23 | 0 | 17 |
| 26 | 25 | 23 | 28 | 12 | 33 | 18 | 23 | 28 | 18 | 30 | 21 | 20 | 0 | 29 |
| 27 | 25 | 19 | 20 | 20 | 31 | 20 | 21 | 25 | 18 | 28 | 18 | 24 | 0 | 25 |
| 28 | 16 | 19 | 15 | 0 | 0 | 0 | 0 | 20 | 20 | 26 | 21 | 25 | 23 | 0 |
| 29 | 22 | 18 | 17 | 0 | 0 | 21 | 20 | 22 | 11 | 25 | 17 | 17 | 20 | 21 |
| 30 | 30 | 19 | 18 | 0 | 0 | 0 | 0 | 25 | 10 | 26 | 0 | 0 | 0 | 0 |
| 31 | 0 | 19 | 20 | 0 | 0 | 17 | 18 | 21 | 13 | 25 | 0 | 0 | 0 | 24 |
| 32 | 28 | 27 | 18 | 0 | 0 | 0 | 0 | 23 | 10 | 25 | 20 | 15 | 0 | 0 |
| 33 | 0 | 18 | 20 | 0 | 0 | 0 | 20 | 20 | 12 | 25 | 0 | 0 | 0 | 12 |
| 34 | 0 | 18 | 18 | 0 | 0 | 15 | 17 | 20 | 13 | 25 | 0 | 0 | 0 | 25 |
| 35 | 30 | 20 | 20 | 0 | 25 | 22 | 23 | 20 | 24 | 26 | 22 | 23 | 22 | 25 |
| 36 | 16 | 0 | 0 | 0 | 0 | 0 | 0 | 21 | 23 | 25 | 21 | 0 | 0 | 8 |
| 37 | 25 | 20 | 20 | 23 | 26 | 22 | 24 | 20 | 18 | 28 | 22 | 25 | 0 | 24 |
| 38 | 26 | 20 | 21 | 0 | 0 | 20 | 24 | 25 | 23 | 28 | 21 | 23 | 25 | 24 |
| 39 | 25 | 21 | 22 | 0 | 0 | 20 | 25 | 24 | 25 | 27 | 23 | 0 | 24 | 26 |
| 40 | 26 | 22 | 24 | 0 | 26 | 19 | 24 | 26 | 16 | 26 | 22 | 24 | 23 | 25 |
| 41 | 25 | 23 | 23 | 11 | 25 | 18 | 25 | 25 | 0 | 27 | 22 | 23 | 0 | 26 |
| 42 | 16 | 25 | 24 | 0 | 0 | 16 | 24 | 24 | 16 | 26 | 24 | 0 | 0 | 25 |
| 43 | 26 | 24 | 23 | 0 | 25 | 14 | 26 | 26 | 0 | 25 | 23 | 24 | 26 | 23 |
| 44 | 25 | 24 | 23 | 0 | 0 | 16 | 27 | 25 | 0 | 24 | 25 | 25 | 0 | 0 |
| 45 | 26 | 25 | 24 | 16 | 24 | 0 | 28 | 26 | 0 | 26 | 23 | 24 | 0 | 25 |

Abbreviations: AMP: Ampicillin; VAN: Vancomycin; TPN: Teicoplanin; ERY: Erythromycin; TET: Tetracycline; CIP: Ciprofloxacin; LEV: Levofloxacin; NIT: Nitrofurantoin; QD: Quinupristin/Dalfopristin; LIN: Linezolid; GEN: Gentamicin; STR: Streptomycin; KAN: Kanamycin; CLR: Chloramphenicol.
